# Burnout and the Psychological Impact among Physiatrists in Saudi Arabia during COVID-19

**DOI:** 10.3390/ijerph18189621

**Published:** 2021-09-13

**Authors:** Ahmad H. Alwashmi, Abdulmajeed A. Alkhamees

**Affiliations:** 1 Department of Orthopedic Surgery, College of Medicine, Qassim University, Buraydah 52571, Saudi Arabia; 2 Department of Medicine, Unaizah College of Medicine and Medical Sciences, Qassim University, Buraydah 52571, Saudi Arabia; A.alkhamees@qu.edu.sa

**Keywords:** burnout, COVID-19, anxiety, depression, physiatry, physical medicine and rehabilitation, Saudi Arabia

## Abstract

Background: Burnout is an emerging critical issue facing specialists and trainees in all disciplines and not particularly studied among physiatry specialists and trainees in Saudi Arabia during the COVID-19 pandemic. Objective: To assess physiatrist burnout, depression, anxiety, and stress during the current COVID-19 pandemic crisis in Saudi Arabia. Design: Cross-sectional study. Setting: By distributing an electronic survey, the researcher assessed burnout using the Maslach Burnout Inventory (MBI) Human Services Survey (HSS) in the midst of the curfew that Saudi authorities imposed. Participants: One hundred one participating trainees, specialists, and consultants. Results: Of the 101 study participants, the majority (73.3%) were between the ages of 24 and 34 years old, with the rest distributed within the age group ranging from 35 to 65 years old. Junior residents represented 34.7%, senior residents 22.8%, physiatrist specialists 26.7%, and consultants 15.8%. The sample included 55.4% males and 44.6% females; 64.4% of the participants were married, 29.7% were still single, and 5.9% were divorced. Among the total group participating, 25.7% were handling COVID-19 patients. In the total participant sample, 80.2% reported experiencing burnout, 10.9% experienced stress, and 22.8% and 6.9% experienced anxiety and depression, respectively. Conclusion: Burnout in Saudi Arabia exists among more than two-thirds of practicing physiatrists in Physical Medicine & Rehabilitation (PM&R), and that did not appear to have a statistically significant influence on stress, anxiety, or depression (*p* > 0.05). The current COVID-19 global pandemic might escalate burnout and influence mental health outcomes. The healthcare authority and administration should take the lead in identifying the challenges, overcoming the obstacles, and optimizing clinician well-being, delivering up-to-date solutions, and promptly checking their effectiveness.

## 1. Introduction

During the Coronavirus disease outbreak of 2019 (COVID-19), the level of stress and demands on practicing physicians increased dramatically within a very short period. Almost all the world’s population was under lockdown, and curfews were ordered, paralyzing schools, workplaces, airports, public transportation, and many other aspects of daily life. The COVID-19 infection mainly involves respiratory symptoms, and death results from acute respiratory distress syndrome [1]. Even before this pandemic, systematic reviews among physical medicine and rehabilitation specialists have widely reported the high prevalence of burnout, its causes, and serious outcomes [2].

Professional burnout, emotional exhaustion, and loss of satisfaction with patient care affect doctors at all stages of their career, from residency trainees to certified specialists. Burnout is an emerging critical issue facing specialists and trainees in all disciplines. Burnout in doctors is linked to serious negative patient outcomes, including higher rates of medical errors and poorer quality of care. It is also linked to negative outcomes for doctors, including substance abuse and suicide [2]. Although burnout is a serious problem, little is known about burnout in specialists and trainees in Physical Medicine & Rehabilitation (PM&R). A systematic review was conducted to understand whether burnout is, in fact, a problem for doctors in PM&R. It reported that more than half of physiatrists reviewed, including specialists and trainees, experience burnout at a higher rate than non-rehabilitation doctors do. Working in PM&R is a unique risk factor for burnout among doctors. Important next steps will include understanding what causes such high rates of burnout and what can be done to help [2].

In Saudi Arabia, physical medicine and rehabilitation specialization, or “physiatry,” is a fast-growing medical specialty, with more than 120 specialists currently practicing and over 40 residents in training. However, no known studies describe burnout among physiatrists in Saudi Arabia, especially during such a crisis as the COVID-19 pandemic.

Studies of previous outbreaks of severe acute respiratory syndrome (SARS), Middle East respiratory syndrome (MERS), influenza, and H1N1 report that all health workers, both physicians and non-physicians, experience burnout to various degrees. Anxiety and stress developing in the physicians during outbreaks were found to have a positive relationship with the Maslach Burnout Inventory [3,4]. Other factors were found to contribute to the development of burnout in addition to the typical contributors, such as lack of control over procedures, the false notion of safety precautions, lack of preparedness and emotional support, infection-control measures, poor communication and directives, insufficient personal protective equipment (PPE), and perceived fatalities [4].

This study aims to assess physiatrist burnout, depression, anxiety, and stress during the current COVID-19 pandemic crisis in Saudi Arabia. A secondary aim is to assess sociodemographic characteristics and their correlation with burnout, depression, anxiety, and stress among physiatrists in Saudi Arabia.

## 2. Materials and Methods

### 2.1. Study Design and Setting

This study was conducted using a cross-sectional design to assess physiatrist burnout, depression, anxiety, and stress during the current COVID 19 pandemic in Saudi Arabia. The study reporting followed the Strengthening the Reporting of Observational Studies in Epidemiology Checklist. 

There are more than 120 practicing specialists and over 40 residents in Saudi Arabia divided into two training locations. The program consists of four training years divided into a junior level (R1–R2) and a senior level (R3–R4) with rotations in inpatient physical medicine and rehabilitation units and outpatient clinics.

All physiatrists (attending and residents) located in Saudi Arabia were included in the sample. They were approached using an online survey sent to WhatsApp groups which contain all physiatrists practicing in Saudi Arabia. Physical distribution was not feasible due to the applied protective measures.

### 2.2. Study Procedure

The survey was distributed in the midst of the curfew that was imposed by Saudi authorities, and at that time the Saudi Ministry of Health suspended all unnecessary appointments and procedures inside all hospitals inside Saudi Ariba.

The survey was performed between 27 May and 8 August 2020, using a Google survey website (Google LLC, Mountain View, CA, USA). The Institutional Review Board of Qassim University approved the study (No.19-10-02). All participants were informed about the study’s purposes and provided informed consent. Data were kept confidential and were not disclosed unless for study purposes. 

### 2.3. Variables and Instruments

The study’s survey includes sociodemographic data about participants (age, gender, parental status, marital status, their current job role, level of the hospital they are currently working at), as well as four statements concerning their current attitudes toward COVID-19.

Burnout was assessed using the Maslach Burnout Inventory (MBI) Human Services Survey (HSS), an assessment test developed by Maslach and Jackson in 1981 to measure personally perceived burnout. [5,6] Using this tool, three domains of burnout were assessed—emotional exhaustion, depersonalization, and personal accomplishment, each with its own subscale. Participants who had scores ≥ 27 on the emotional exhaustion subscale, ≥13 on the depersonalization subscale, or ≤31 on the personal accomplishment subscale were considered to have experienced burnout symptoms. Those who scored ≥27 on the emotional exhaustion subscale and/or ≥13 on the depersonalization subscale were considered to be suffering from burnout. When tested for their reliability, each of the subscales of the MBI performed well, with the Cronbach’s alpha of the EE, DP, and PA subscales being 0.91, 0.81, and 0.72, respectively [7,8].

Stress, anxiety, and depression were assessed using corresponding subscales on the DASS-21 assessment tool. Each subscale contains seven items, with each response rated from zero to three, where zero implies ‘Did not apply to me’ and three implies ‘Applied to me most of the time’ [9]. 

The Depression subscale was assessed in items 3, 5, 10, 13, 16, 17, and 21. The total score depression subscale score was subdivided into normal (0–9), mild (10–12), moderate (13–20), severe (21–27), and extremely severe depression (28–42) [10]. The anxiety subscale was assessed in items 2, 4, 7, 9, 15, 19, and 20. The total score of the anxiety subscale was subdivided into normal (0–6), mild (7–9), moderate (10–14), severe (15–19), and extremely severe anxiety (20–42) [10]. The stress subscale was composed of items 1, 6, 8, 11, 12, 14, and 18. The total score of the stress subscale was subdivided into normal (0–10), mild (11–18), moderate (19–26), severe (27–34), and extremely severe stress (35–42) [10].

All three subscales were found to have strong reliability based on Cronbach’s alphas for each subscale (DASS stress subscale: 0.77, DASS anxiety subscale: 0.71, and DASS depression subscale: 0.76).

### 2.4. Statistical Analysis

A total of 101 participants were recruited into the study and requested to provide data on their experiences with burnout and resulting mental health outcomes of stress, anxiety, and depression. The data collected was first entered into a Microsoft Excel spreadsheet for cleaning and validation, prior to being transferred to a spreadsheet on IBM SPSS Statistics for Windows, version 22.0 (IBM Corp., Armonk, NY, USA) for analysis. Descriptive analyses were carried out to describe the variables, with the results presented as frequencies and percentages for categorical data, and mean with standard deviation for continuous data. Inferential statistics were conducted to determine the presence and magnitude of associations between sociodemographic/occupational variables and burnout/mental health outcomes. Chi-squared tests (with appropriate Fisher’s exact tests where necessary) were run to determine relationships between variables. All tests were done at a level of significance set at *p* < 0.05.

## 3. Results

### 3.1. Sociodemographic and Educational Characteristics

Table 1 describes the different Sociodemographic and Educational Characteristics among participants.

Looking closely at the current attitudes of the participants with regards to COVID-19, about 78.2% of the participants were worried about becoming infected with the disease. Slightly less than that indicated that they feel more burnout now than before COVID-19 (65.3%). When asked if they felt worried about any member of their family being infected, 22.8% of the participants agreed, and 72.3% strongly agreed. Almost all the participants worried about the crisis going on for too long (92.1%). (Table 2).

### 3.2. Prevalence of Burnout and Depression among the Study Population

As presented in Table 3, 80.2% of the participants experienced burnout which was determined based on their scoring high on the emotional exhaustion and/or the depersonalization subscales of the MBI-HSS. 

Table 4 describes the severity of the mental health outcomes of stress, anxiety, and depression experienced by the study participants. 

### 3.3. Relationship between Burnout and Mental Health Outcomes among the Study Population

Associations between independent variables (sociodemographic and occupational characteristics) and burnout/mental health outcomes yielded results presented in Table 5. With regards to burnout, gender, type of hospital, and current roles were the variables that had statistically significant associations with the experience of burnout. 91.1% of females (vs. 71.4% of males) experienced burnout, indicating that female physiatrists are more likely to experience burnout than their male counterparts (*p* = 0.014). Physiatrists who worked in tertiary centers are more likely to experience burnout, as 83.1% of physiatrists who worked in such centers experienced burnout in this study as compared to 58.3% of those who worked in primary/secondary care centers who did (*p* = 0.043). Physiatrists in-training (junior and senior residents) are more likely to experience burnout than their senior counterparts who are specialists or consultants (*p* = 0.006).

Concerning the mental health outcomes of burnout among the study participants, a number of the participants experienced stress, anxiety, and/or depression. Burnout itself did not appear to have a statistically significant influence on either stress, anxiety, or depression even though participants who had experienced burnout were more likely to experience any one of these symptoms (*p* > 0.05). With regard to stress, participants’ current job role/designation and whether or not they have been handling COVID-19 patients had a significant influence on the expression of stress. Residents (junior or senior) were more likely to experience stress than their more senior counterparts, who are specialists or consultants (*p* = 0.022). Those who were also involved in the care of COVID-19 patients were significantly more likely to experience stress (*p* = 0.021).

With regard to anxiety, only one of the factors had a statistically significant influence on the expression of anxiety–handling COVID-19 patients. As much as 38.5% of the participants involved in the handling of COVID-19 patients experienced anxiety when compared with 17.3% of participants not involved in the handling of COVID-19 patients who experienced anxiety, suggesting that physiatrists involved in the handling of COVID-19 patients were more likely to suffer from anxiety (*p* = 0.027). None of the factors tested had any statistically significant influence on depression (*p* > 0.05).

## 4. Discussion

Currently, limited evidence exists of burnout and resulting mental health outcomes of stress, anxiety, and depression among physiatry specialists and trainees [2]. In Saudi Arabia, as far as the authors know, no prior studies conducted among practicing physiatrists have examined the effects of the current pandemic. Our results show that 80.2% of participants experienced burnout, determined by their high scores on the emotional exhaustion and/or depersonalization subscales of the MBI-HSS. In contrast, a recent study in the United States measured the exposure to COVID-19 patients and the possibility of developing burnout and stress among physician trainees. It found that, compared to the non-exposed group, the exposed group had a higher prevalence of stress (29.4% vs. 18.9%) and burnout (46.3% vs. 33.7%) [11]. Another recent study in India measuring burnout among healthcare workers during the COVID-19 pandemic found an increase in personal burnout of 44.6%, where work-related burnout was 26.9%—that is, compared to normal circumstances, pandemic-related burnout represents a significant increase [12]. Moreover, a recent systematic review investigated the prevalence of burnout among PM&R physicians and found emerging evidence that PM&R physicians are among the most likely to experience burnout [2], not to mention the added effect of the current global pandemic. However, burnout itself did not appear to have a statistically significant influence on stress, anxiety, or depression, even though participants who had experienced burnout were more likely to experience any one of these symptoms. 

When left unaddressed, burnout is progressive and can affect both patient and caregiver safety and wellbeing. Experts have suggested that burnout could relate to systemic factors rather than to the physician’s personal resilience [13]. Scientific journals specific to rehabilitation medicine have featured diverse opinions on burnout, ranging from acknowledging that PM&R physicians are at increased risk by the nature of the work they do [14], to criticizing burnout as a legitimate condition threatening physician well-being and viewing it as a “myth” [15], to describing the prevalence of burnout as a research priority [16]. However, a systematic review presents evidence that strongly challenges the belief that burnout among physiatrists is a myth; it emphasizes the need for additional research to evaluate the prevalence of burnout in PM&R physicians [2]. The high percentage in our results could be attributed to the fact that 78.2% of the participants were worried about becoming infected with the disease, and 65.3% reported feeling burnout more than ever before. Regarding burnout and gender, our results showed that 91.1% of females (as opposed to 71.4% of males) experienced burnout, indicating that female physiatrists are more likely to experience burnout than their male counterparts. Many studies have found female physicians to have a 20–60% greater chance of exhaustion, compared to males [17]. Females might be more susceptible to developing burnout due to the strong influence of emotional exhaustion on depersonalization, which might result in low levels of personal accomplishment [18]. Moreover, a study from Norway reported the predictive value of individual factors, work-related factors, and work–home interaction on burnout among female and male physicians. Much higher levels of exhaustion among women resulted from work–home disputes, whereas workload was the strongest burnout predictor in male physicians [19].

Concerning the mental health outcomes, 10.9% of our participants experienced stress, with 22.8% and 6.9% experiencing anxiety and depression, respectively. In comparison, our result was lower than that of a recent study conducted among healthcare workers during the COVID-19 outbreak in Saudi Arabia, which reports 51.4% and 55.2% of their sample, consisting of both physicians and non-physicians, suffered from generalized anxiety disorder and depression, respectively. This difference in results could be attributed to the different samples and instruments used by the two studies. [20].

Junior or senior residents were more likely to experience stress than their colleagues who are specialists or consultants, and this finding aligns with other studies conducted even before the current pandemic. Residents usually must struggle with balancing the roles of learner and caregiver, particularly during the current pandemic in which high levels of stress have emerged [11]. Those also involved in the care of COVID-19 patients were significantly more likely to experience stress. Participants involved in the handling of COVID-19 patients experienced anxiety at the rate of 38.5%, consistent with a recent study conducted in the United States, which found that trainees exposed to COVID-19 patients had more stress than the non-exposed group. The high level of stress that this pandemic creates and the fear of becoming infected and infecting family members, as more than 80% of our sample reported, could explain this finding [11].

Only about a quarter of the participants answered in the affirmative to the question of whether they were handling COVID-19 patients (25.7%), a relatively small percentage that was expected since most of the rehabilitation physicians are not front-line physicians such as emergency and ICU physicians. However, during the progression of COVID-19, there was a sudden surge in need for medical care. With this surge in demand, a shortage in medical staff surfaced, leading to the need for physicians not on the front line, such as physiatrists, to come on the scene.

Regarding the type of hospital where the participants worked, as we expected, the majority worked in tertiary care facilities (88.1%), owing to the location of the PM&R residency programs in Saudi Arabia, where a large percentage of the study subjects are junior (34.7%) and senior (22.8%) residents, while the rest work in either primary (2.0%) or secondary care facilities (9.9%). Physiatrists who work in tertiary care centers were more likely to experience burnout; 83.1% of physiatrists who worked in such centers experienced burnout, compared with 58.3% of those who did so while working in primary/secondary care centers. In Saudi Arabia, most of the primary-rehabilitation and secondary care facilities do not offer inpatient services rather than outpatient rehabilitation services. The necessity for inpatient admission would trigger initiating a referral to a tertiary care rehabilitation center, where practice includes increased workloads, long-duty working hours, numerous on-call duties (weekdays and weekends), meticulous documentation in electronic medical records, and continuing uncompleted work-related assignments at home. Without a doubt, workload and demand in tertiary healthcare centers are greater than those in primary and secondary healthcare centers, increasing the odds that burnout will develop.

There is no consensus on how best to prevent or manage burnout. To date, the authors are not aware of any studies of prevention or treatment of burnout in specialists or trainees in PM&R. Previous recommendations range from individual and organizational interventions [21,22] to focusing on physiatrists’ mission [16]. On an individual level, PM&R specialists and trainees should receive tools to understand, prevent, identify, and treat burnout. Establishing relevant core competencies in residency training and continuing medical education resources for specialists are two important ways to start. However, placing the burden solely on individuals and considering burnout as an individual problem ignore the larger context in which burnout develops.

In reality, physicians continue to experience burnout, and residents in training are not doing better, according to previous studies [22]. In addition to the recent pandemic, where physicians are expected to be at their best and the level of burnout might increase, addressing burnout should shift toward being a responsibility shared between physicians and the healthcare system. Launching initiatives to empower and support residents as they take responsibility for their well-being would be best. In their meta-analysis [22], Kimberly et al. report that job training/education was the most effective organizational intervention. Other studies suggest that training and prevention processes can reduce burnout incidence, and combining organizational changes with work environment change can have more significant effects [22].

Healthcare authorities and administrations should take the lead in identifying the challenges and obstacles to overcome, optimizing clinician well-being, delivering up-to-date solutions, and checking their effectiveness promptly. That may include regular surveys, using numerous established scales that quickly measure the physical, emotional, and mental exhaustion of the practicing clinicians. Physicians surrounded by many mortality and morbidity situations in PM&R, in particular, are encircled by patients, most of whom have lost a massive part of their health and well-being, such as losing the ability to walk, talk, or even eat, and chances of mental and emotional exhaustion are high. Scheduled breaks and days off should be offered and carefully planned. Also, physicians should be encouraged to enroll in and be a part of relaxation techniques, such as yoga and meditation, and approach therapists to vent emotions and frustrations. That might help them to return to work with a more relaxed and balanced mindset, to increase work productivity more efficiently.

## 5. Limitations

This cross-sectional survey study has potential limitations. The small sample size and the subpopulation of the study in which data were collected represented one subspecialty in one country, indicating an important need to expand the scope of research to different specialties around the world. Furthermore, the data collection occurred during the early period of the pandemic, when people were under 24-h curfews, so this might not represent the current state of burnout and anxiety. Moreover, additional factors affecting the level of burnout anxiety, like personality traits, job-related factors, and level of support from the organization and family, might be considered. Also, some authors in the literature have suggested that the DASS has difficulty in properly identifying and discriminating between symptoms associated with depression and anxiety. Lastly, important future research should investigate the lasting affection effect of the psychological symptoms after the immediate threat of the pandemic subsides.

## 6. Conclusions

Burnout in Saudi Arabia exists among more than two-thirds of practicing physiatrists/PM&R physicians and did not appear to have a statistically significant influence on stress, anxiety, or depression. The current COVID-19 global pandemic might escalate burnout and influence mental health outcomes. Healthcare authorities and administrations should take the lead in identifying and overcoming the challenges and obstacles, optimizing clinician well-being, delivering up-to-date solutions, and checking their effectiveness promptly.

## Figures and Tables

**Table 1:** Sociodemographic characteristics of the study population.

Variables	Frequency	Percent
**Age**		
24–34 years	74	73.3
35–45 years	12	11.9
46–56 years	7	6.9
56–65 years	8	7.9
**Gender**		
Male	56	55.4
Female	45	44.6
**Marital status**		
Single	30	29.7
Married	65	64.4
Divorced	6	5.9
**Raising children**		
Yes	55	54.5
No	46	45.5
**Hospital type**		
Primary care	2	2.0
Secondary care	10	9.9
Tertiary care	89	88.1
**Working hours**		
≤40 h/week	28	27.7
41–60 h/week	70	69.3
61–80 h/week	3	3.0
**Current role**		
Junior resident	35	34.7
Senior resident	23	22.8
Specialist	27	26.7
Consultant	16	15.8
**Handling COVID-19 patients**		
Yes	26	25.7
No	75	74.3
**Received mental health help in the past year**		
Yes	6	5.9
No	95	94.1

**Table 2:** Current attitudes toward COVID-19 among the study population.

Statements	Strongly Disagree	Disagree	Neutral	Agree	Strongly Agree
Worried about becoming infected	2 (2.0%)	−	20 (19.8%)	28 (27.7%)	51 (50.5%)
Feel more burnout now than before COVID-19	9 (8.9%)	12 (11.9%)	14 (13.9%)	26 (25.7%)	40 (39.6%)
Worried about family becoming infected	3 (3.0%)	1 (1.0%)	1 (1.0%)	23 (22.8%)	73 (72.3%)
Worried about the crisis going on for too long	−	−	8 (7.9%)	30 (29.7%)	63 (62.4%)

**Table 3:** Prevalence of burnout and mental health outcomes among the study population (*n* = 101).

Variables	Frequency	Percent (%)
Burnout syndrome *		
Yes	81	80.2
No	20	19.8
**Burnout subscales**		
High emotional exhaustion	52	51.5
High depersonalization	72	71.3
Low personal accomplishment	92	91.1
Stress **		
Yes	11	10.9
No	90	89.1
Anxiety **		
Yes	23	22.8
No	78	77.2
Depression **		
Yes	7	6.9
No	94	93.1

* Determined based on respondents having a high score on the emotional exhaustion and/or depersonalization subscales (see methods). ** Stress, anxiety, and depression were determined using the appropriate subscales on the DASS-21.

**Table 4:** Participants’ performance on the three subscales of the Depression, Anxiety, and Stress Scale—21 Items (DASS-21).

	Stress *	Anxiety *	Depression *
Score, Mean ± SD	10.5 ± 6.2	5.0 ± 5.4	6.0 ± 5.8
Categories, *N* (%)			
Normal	74 (73.3%)	67 (66.3%)	74 (73.3%)
Mild	16 (15.8%)	11 (10.9%)	20 (19.8%)
Moderate	10 (9.9%)	16 (15.8%)	5 (5.0%)
Severe	1 (1.0%)	5 (5.0%)	1 (1.0%)
Extremely severe	−	2 (2.0%)	1 (1.0%)

* subscales of the DASS.

**Table 5:** Association between sociodemographic variables and each of burnout, stress, anxiety, and depression (*n* = 101).

Variables	Burnout			Stress			Anxiety			Depression		
	Yes	No	*p*	Yes	No	*p*	Yes	No	*p*	Yes	No	*p*
**Age**												
24–34 years	61 (82.4%)	13 (17.6%)	0.351	10 (13.5%)	64 (86.5%)	0.280	19 (25.7%)	55 (74.3%)	0.296	7 (9.5%)	67 (90.5%)	0.098
35–65 years	20 (74.1%)	7 (25.9%)		1 (3.7%)	26 (96.3%)		4 (14.8%)	23 (85.2%)		0 (0.0%)	27 (100.0%)	
**Gender**												
Male	40 (71.4%)	16 (28.6%)	0.014	5 (8.9%)	51 (91.1%)	0.480	12 (21.4%)	44 (78.6%)	0.719	3 (5.4%)	53 (94.6%)	0.697
Female	41 (91.1%)	4 (8.9%)		6 (13.3%)	39 (86.7%)		11 (24.4%)	34 (75.6%)		4 (8.9%)	41 (91.1%)	
**Marital status**												
Single/divorced	31 (86.1%)	5 (13.9%)	0.267	5 (13.9%)	31 (86.1%)	0.472	8 (22.2%)	28 (77.8%)	0.922	4 (11.1%)	32 (88.9%)	0.244
Married	50 (76.9%)	15 (23.1%)		6 (9.2%)	59 (90.8%)		15 (23.1%)	50 (76.9%)		3 (4.6%)	62 (95.4%)	
**Raising children**												
Yes	41 (74.5%)	14 (25.5%)	0.119	5 (9.1%)	50 (90.9%)	0.525	14 (25.5%)	41 (74.5%)	0.482	3 (5.5%)	52 (94.5%)	0.699
No	40 (87.0%)	6 (13.0%)		6 (13.0%)	40 (87.0%)		9 (19.6%)	37 (80.4%)		4 (8.7%)	42 (91.3%)	
**Hospital type**												
Primary/Secondary care	7 (58.3%)	5 (41.7%)	0.043	2 (16.7%)	10 (83.3%)	0.616	5 (41.7%)	7 (58.3%)	0.096	2 (16.7%)	10 (83.3%)	0.194
Tertiary care	74 (83.1%)	15 (16.9%)		9 (10.1%)	80 (89.9%)		18 (20.2%)	71 (79.8%)		5 (5.6%)	84 (94.4%)	
**Working hours**												
≤40 h/week	19 (67.9%)	9 (32.1%)	0.054	3 (10.7%)	25 (89.3%)	0.972	5 (17.9%)	23 (82.1%)	0.466	3 (10.7%)	25 (89.3%)	0.393
41–80 h/week	62 (84.9%)	11 (15.1%)		8 (11.0%)	65 (89.0%)		18 (24.7%)	55 (75.3%)		4 (5.5%)	69 (94.5%)	
**Current role**												
Junior/senior resident	52 (89.7%)	6 (10.3%)	0.006	10 (17.2%)	48 (82.8%)	0.022	15 (25.9%)	43 (74.1%)	0.390	6 (10.3%)	52 (89.7%)	0.234
Specialist/consultant	29 (67.4%)	14 (32.6%)		1 (2.3%)	42 (97.7%)		8 (18.6%)	35 (81.4%)		1 (2.3%)	42 (97.7%)	
**Handling COVID-19 patients**												
Yes	23 (88.5%)	3 (11.5%)	0.220	6 (23.1%)	20 (76.9%)	0.021	10 (38.5%)	16 (61.5%)	0.027	3 (11.5%)	23 (88.5%)	0.369
No	58 (77.3%)	17 (22.7%)		5 (6.7%)	70 (93.3%)		13 (17.3%)	62 (82.7%)		4 (5.3%)	71 (94.7%)	
**Received mental health help**												
Yes	6 (100.0%)	0 (0.0%)	0.209	1 (16.7%)	5 (83.3%)	0.509	1 (16.7%)	5 (83.3%)	0.713	1 (16.7%)	5 (83.3%)	0.358
No	75 (78.9%)	20 (21.1%)		10 (10.5%)	85 (89.5%)		22 (23.2%)	73 (76.8%)		6 (6.3%)	89 (93.7%)	
**Burnout**												
Yes	−	−	NA	10 (12.3%)	71 (87.7%)	0.688	19 (23.5%)	62 (76.5%)	0.741	5 (6.2%)	76 (93.8%)	0.623
No	−	−		1 (5.0%)	19 (95.0%)		4 (20.0%)	16 (80.0%)		2 (10.0%)	18 (90.0%)	

Fisher’s exact test (all others are chi-squared tests). Bolded: *p*-values are significant.

## Data Availability

The data that support the findings of this study are available from the corresponding author, [A.H.A.], upon reasonable request.
